# Predictive role of [^18^F]FDG PET-CT radiomic parameters for KRAS/BRAF/EGFR mutations in metastatic colorectal cancer patients

**DOI:** 10.1186/s41824-024-00233-5

**Published:** 2024-12-26

**Authors:** Magdi A. Ali, Omar Shebl Zahra, Mohmed I. Morsi, Mohamed M. El Safwany, Shaymaa Essam El Feky

**Affiliations:** 1 Faculty of Health Sciences, Gulf Medical University, Ajman, United Arab Emirates; 2 Radiation Sciences Department, Medical Research Institute, University of Alexandria, Alexandria, Egypt; 3 Clinical Oncology and Nuclear Medicine Department, Faculty of Medicine, University of Alexandria, Alexandria, Egypt; 4 Radiology and Medical Imaging Department, Faculty of Applied Health Sciences Technology, Pharos University, Alexandria, Egypt

**Keywords:** Metastatic colorectal cancer, Mutations, [^18^F] FDG, PET-CT, Radiomics

## Abstract

**Objectives:**

The objective of this study was to evaluate the predictive value of ^18^F-fluorodeoxyglucose [^18^F]FDG positron emission tomography (PET-CT) radiomic parameters in relation to KRAS/BRAF/EGFR mutations in patients with metastatic colorectal cancer (mCRC).

**Methods:**

Blood samples were collected from 90 mCRC patients to assess KRAS G13V, BRAF V600E, and EGFR exon 20 mutations. [^18^F]FDG PET-CT scans were performed, and radiomic parameters, including the SUV max, max TBR, total MTV, and total TLG, were calculated and correlated with different genotypes and haplotypes of the aforementioned mutations.

**Results:**

The SUV max, TLG, and TBR were significantly greater in patients with the KRAS G13V and BRAF V600E mutations than in patients with the wild-type genotype. The SUVmax was also significantly greater in patients with EGFR exon 20 mutations. Haplotype analysis revealed that the SUVmax was significantly greater in patients with KRAS/BRAF/EGFR mutations than in other patients, with a specificity of 68.18% and sensitivity of 65.28%.

**Conclusion:**

The results suggest that [^18^F] FDG PET-CT radiomic parameters, particularly the SUV max, have the potential to serve as noninvasive tools for predicting the KRAS/BRAF/EGFR mutation status in mCRC patients.

## Introduction

Colorectal cancer (CRC) is the second most deadly cancer and the third most commonly diagnosed cancer worldwide, representing one in ten cancer cases and deaths (Klimeck et al. 2023).

The development of CRC is a result of several genetic abnormalities, including genetic mutations, epigenetic modifications, and chromosomal aberrations, that affect cell proliferation, differentiation, and programmed cell death. It is now generally acknowledged that preneoplastic lesions often trigger the activation of oncogenes and the inactivation of tumor suppressor genes and mismatch repair genes, leading to sporadic colorectal cancers (Centelles 2012; Alfahed 2023).

The limitations of colonoscopy have driven the need for the development of non-invasive or minimally-invasive biomarker tests that can be used for CRC screening and early detection. However, there are currently few molecular biomarkers available for CRC, and those that do exist, such as carcinoembryonic antigen (CEA), have limited utility due to poor specificity(xxx. xxxx).

There is a pressing need for the development of novel, noninvasive biomarkers that can enhance the early diagnosis of CRC, monitor disease progression, and guide personalized treatment. Recent advancements in translational research have yielded a wide range of potential CRC biomarkers, including genetic, epigenetic, and metabolomics alterations. These biomarkers hold promise for improving CRC screening, detection, and management.(yyy. xxxx).

Biomarkers can serve various purposes in the management of mCRC, including early detection, prognostic assessment, treatment selection, and monitoring of disease progression or response to therapy. However, the clinical utility of biomarkers in mCRC has been variable, with some established biomarkers, such as RAS and BRAF mutation status, while others remain under investigation (zzz. xxxx).

In recent years, significant progress has been made in understanding the molecular mechanisms underlying CRC development and progression. Genetic and epigenetic alterations in key signaling pathways, such as the RAS/MAPK and PI3K/AKT pathways, have been identified as major drivers of CRC carcinogenesis. These insights have led to the development of a new class of targeted therapies known as tyrosine kinase inhibitors (TKIs), which have shown promising results in the treatment of CRC (aaaa. xxxx).

Epidermal Growth Factor Receptor (EGFR) is a crucial component of the mitogen-activated protein kinase (MAPK) pathway. Mutations that activate EGFR can cause abnormal cell division, reduced apoptosis, angiogenesis, enhanced cell survival, and gene transcription (Choi et al. 2023; Wee and Wang 2017). EGFR aberration leads to the overexpression of downstream pro-oncogenes regulating several pathways, including the RAS-RAF-MEK-ERK MAPK and AKT-PI3K-mTOR pathways. Kirsten Rat Sarcoma Viral Oncogene Homolog (KRAS) is the most commonly mutated gene among the three human RAS isoforms; mutations are found in 17–25% of all malignancies. In particular, KRAS mutations are present in between 30 and 40% of colon cancer cases (Li et al. 2023; Aguilera and Serna-Blasco 2018). Mutations in exon 2 at codons 12 and 13 represent 90% of KRAS mutations in CRC and have been linked to worse prognoses and more aggressive tumors (Lian, et al. 2023; Pritchard and Grady 2011). Downstream of EGFR and KRAS, the protein kinase encoded by the V-Raf murine sarcoma viral oncogene homolog B1 (BRAF) gene is affected by mutations detected in 5–15% of colorectal cancer patients (Aguilera and Serna-Blasco 2018). V600E BRAF is the most common BRAF mutation and is associated with worse clinical outcomes and shorter overall survival (Sinicrope 2019). Although several therapeutic agents, including monoclonal antibodies against EGFR, have been developed in recent years as first-line treatments, mutations in KRAS and BRAF have been reported to render anti-EGFR therapies ineffective (Bellio et al. 2021).

Fluorodeoxyglucose positron emission tomography computed tomography [^18^F]FDG-PET/CT is particularly useful for diagnosing, staging, and tracking therapeutic response in oncology imaging (Ali et al. 2023).

PET-CT is commonly utilized to detect metastases in CRC patients. Many studies have focused on quantifying PET-CT and treatment outcomes. The extraction of image features and their associations with patient mutational status could be useful tools for predicting treatment outcomes (Cho et al. 2017; Lee et al. 2023). In many clinical studies, the concentration of [^18^F]FDG was relatively high in CRC tumors with KRAS and BRAF mutations (He, et al. 2021). This relationship may have prognostic significance for poor prognosis, and our findings may aid in the accurate treatment of CRC patients (Arslan, et al. 2020).

Given these limitations of current diagnostic methods, our study emphasizes the need for more innovative strategies to improve CRC detection, particularly in advanced or metastatic disease.

The purpose of this study was to assess the ability of metabolic parameters, radiomic features, PET-CT, and clinicopathological data to detect EGFR, BRAF, and KRAS mutation status in metastatic colorectal cancer patients (mCRC). To our knowledge, this is the first clinical study demonstrating an association between EGFR, BRAF, and KRAS and [^18^F] FDG accumulation in metastatic CRC. Our study suggested that radiomic features from PET-CT scans may be useful for predicting EGFR, BRAF, and KRAS mutations in mCRC.

## Materials and methods

### Patients

A total of 90 mCRC patients were selected from among those referred to the Ayadi Al-Mostakbal Oncology Center in Alexandria, Egypt, between June 2021 and February 2022. The study was approved by the ethical committee of the Medical Research Institute (IRB-MRI-STD-9-August-2020), University of Alexandria, Egypt, and informed written consent for patients' participation in clinical research was obtained before inclusion in the study protocol according to ethical guidelines. Clinical history data were collected for all participants, as well as a regular assessment of clinical pathology reports and a review of past [^18^F] FDG PET-CT examinations.

Sample size was calculated using Power Analysis and Sample Size Software (PASS 2020) “NCSS, LLC. Kaysville, Utah, USA, ncss.com/software/pass”. Based on previously published research by Peng. et al. in 2021, the mutation rate of KRAS/NRAS/BRAF was 54.12% (46/85) in the studied colorectal cancer cases. Thus, the minimal total hypothesized sample size of 90 eligible metastatic colorectal cancer patients is needed to evaluate the predictive value of 18F-FDG PET/CT radiomic parameters regarding KRAS/BRAF/EGFR mutations in metastatic colorectal cancer (mCRC); taking into consideration 95% level of confidence, assumed effect size of 54%, and precision of 5% using z test (He et al. 2021; Muralidharan 2014).

### Sampling and cell-free DNA extraction

A single venous blood sample was withdrawn (3 ml) from each mCRC patient prior to injection of [^18^F] FDG. The samples were centrifuged for 10 min at 2000 × g to separate the plasma. DNA was then extracted according to the manufacturer’s instructions using Qiagen’s QIAamp® Circulating Nucleic Acid Kit (Hilden, Germany, cat# 55,114). The volume of the plasma was 0.5 mL, and the elution volume was 50 μL. DNA concentration and purity were determined spectrophotometrically at 230, 260, and 280 nm by using a Nano Drop (R) ND-1000 UV‒Visible Spectrophotometer (Thermo Fischer Scientific, USA). The samples were stored at –80 °C until further use.

### Detection of EGFR, BRAF, and KRAS mutations by qPCR

The detection of KRAS (G13V rs112445441 (G > A)), BRAF (V600E rs1133488022 (T > A)), and EGFR (exon 20 rs1050171 (A > G)) mutations was carried out using predesigned TaqMan SNP genotyping assay kits (Thermo Fisher Scientific, USA, cat# 4,351,379). Reactions were carried out in a total volume of 10 µl containing 5 µl of TaqMan Master Mix, 0.5 µl of assay working stock, and 2 µl of sample containing 2 ng of DNA, and the volume was adjusted to 10 µl by adding nuclease-free water. A thermal cycler (Bio-Rad, USA) was used under the following conditions: 95 °C for 10 min, followed by 40 cycles of 15 s at 95 °C and 1 min, at 60 °C. The data were analyzed according to the Fam/Vic dye detection patterns where the Vic probe indicates the wild-type allele, while the FAM probe indicates the mutant allele (Zhou et al. 2018).

### PET imaging and analysis

[^18^F]FDG PET-CT scans were performed using a combined PET-CT scanner (Siemens Biograph True Point 64; Siemens Healthcare, Erlangen, Germany). Patients were asked to fast for at least 4 h, and plasma glucose levels were checked and confirmed to be less than 150 mg/dL. Patients with chronic diseases known to affect [^18^F] FDG absorption, such as, infection and inflammation diabetes, were excluded from the study Imaging was performed 60 min after intravenous injection of 3.7 MBq/kg body weight of [^18^F] FDG. Patients were placed in a supine position with both arms up, and normal breathing was maintained during the scan. Real-time anatomic exposure control was used to reduce the CT radiation dosage by adapting the scan exposure settings to the anatomy of the patient. The CT images were obtained with Pitch = 0.984, 120 kV /50 mA-Care dose; 5 mm collimation, a 0.5 s rotation period, and a B30f convolution kernel. A CT scan of the brain, neck, chest, abdomen, pelvis, and proximal femurs was conducted without I.V. contrast prior to PET imaging for attenuation correction and anatomical localization. The reconstruction was carried out with a 1.2 mm section thickness. Following CT, PET data were obtained with an acquisition time of 2 min for 5–7 various bed positions (Sagara et al. 2021). [^18^F]FDG PET images were reconstructed and attenuation corrected by CT images then Digital Imaging and Communications in Medicine (DICOM) images were extracted from PETCT scan and sent to workstation. For quantitative analysis, two experienced radiologists/nuclear medicine physicians independently assessed [^18^F] FDG accumulation using the LIFEx package workstation (version 7.3.0; http://www.lifexsoft.org). Image segmentation was performed using the LIFEx software. The reviewers had no prior knowledge of the mutational status of the patients. The study assessed several radiomic measures, including Standard Uptake Values (SUV), metabolic tumor volume (MTV), total lesion glycolysis (TLG), and tumor-to-background ratios (TBR). These parameters were measured across multiple lesions, with each patient having between 3 and 10 lesions.

1. **SUV** it measures normalized radioactivity concentration in PET images using the following equation: SUV = activity concentration in tissue/(injected activity/body weight). To determine the SUV for a tumor, a region of interest (ROI) is centrally placed within the target, and the SUV is calculated automatically based on the observed radioactivity normalized to the average body radioactivity. The SUV can be reported in three ways: SUVmean (the average SUV across all voxels in the ROI, which is less sensitive to image noise but can vary with ROI design and observer differences), SUVmin (the lowest voxel value within the ROI), and SUVmax (the highest voxel value within the ROI) For mCRC evaluation, the highest SUV in primary and metastatic tumors was taken as the SUVmax.
2. **MTV**, a semi-quantitative measure of tumors metabolic activity based on 18F-FDG PET/CT images. MTV might be used to assess tumors biology, treatment response, and prognosis in a variety of cancers. MTV was calculated automatically by an interactive workstation as the sum of the primary and metastatic tumors metabolic volumes, with a threshold of 2.5 SUVmax, as evidenced by the healthy liver tissue region. MTV measures the active volume in milliliters (mL) (Houseni et al. 2021). The MTV has been automatically measured with threshold settings ((absolute SUV threshold = SUV = 2.5), (SUV/ liver activity ratio = 1.5), (% of SUV max threshold = 41%), (adaptive threshold-Beta = 0.30), (contrast based threshold enabled)) using the LIFEx package workstation (version 7.3.0; http://www.lifexsoft.org) using the LIFEx package workstation (version 7.3.0; http://www.lifexsoft.org).
3. **TLG**, a semiquantitative measure, denotes the cell mass of the target lesion (primary and metastatic tumors) that is calculated by multiplying the SUVmean by the MTV. The resulting unit for TLG is SUV*mL (Kaymak et al. 2021)
4. **TBR**, were determined using the equation TBR = SUVmax (lesion)/SUVmean (liver), where each reconstruction technique calculates the SUVmax of lesions and the SUVmean of healthy liver tissue (Tan et al. 2022).

After that, the images were examined for evaluation on the LIFEx package workstation (version 7.3.0; http://www.lifexsoft.org). After independently reviewing all of the PET-CT data, two seasoned nuclear medicine radiologists agreed on the findings. The reviewers' knowledge of the KRAS, BRAF, and EGFR mutational status was concealed.

### Statistical analysis

The data were analyzed using the Statistical Analysis in Social Science (SPSS) software package version 20.0. (IBM Corporation, Chicago, Illinois, USA). Quantitative data are presented as the means and standard deviation. The normality of the quantitative variable distributions was checked using the Kolmogorov‒Smirnov test. The Mann‒Whitney test was used when comparing two groups, and the Kruskal‒Wallis test was used when comparing more than two groups. Receiver operating characteristic (ROC) curves were used to examine the sensitivity and specificity of the SUV max, TBR, and TLG in predicting mutations in mCRC patients. In all the statistical studies, a p value of 0.05 indicated statistical significance.

## Results

### Study population

Ninety mCRC patients were enrolled in this study, 41 of whom were male (45.6%) and 49 of whom were female (54.4%). The patients’ ages ranged from 55 to 84 years, with a mean value of 57.34 ± 12.15 years. Seventy-five patients had primary lesions in the colon (83%), twelve had primary rectal lesions (12%), and three had primary rectal lesions in the rectosigmoid (3%).

### Descriptive analysis of KRAS, BRAF and EGFR mutations in mCRC patients

The genotypic and allelic frequencies of the three variants are presented in Table 1. The distribution of the three variants followed the Hardy‒Weinberg equilibrium (p = 0.109, 0.067, and 0.085 for the studied BRAF, KRAS, and EGFR mutations, respectively). The distribution of the BRAF V600E mutation showed that the genotype of the majority of mCRC patients was mutant (71.1%), while the rest were heterozygous; therefore, all studied patients were represented in the dominant model. For the KRAS G13V mutation, 50% of the included patients had a mutant genotype, 46.7% were carriers of the mutation, and 3.3% had a wild-type genotype; thus, the dominant model represented 96.7% of the included patients. The EGFR (A > G) mutation was the least frequent, as 17.8% of patients had a mutant genotype, 58.9% had a heterogeneous genotype, and the rest had a wild-type genotype. The dominant model of EGFR (A > G) mutation represented 76.7% of the studied population.

**Table 1:** Distribution of the studied patients according to different SNPs and models

	No	%
Individual SNPS		
BRAF V600E (rs1133488022)		
TT	0	0.0
TA	26	28.9
AA	64	71.1
T	26	14.4
A	154	85.6
KRAS G13V (rs112445441)		
GG	3	3.3
GA	42	46.7
AA	45	50.0
Allele		
G	48	26.7
A	132	73.3
EGFR exon 20 (rs1050171)		
AA	21	23.3
AG	53	58.9
GG	16	17.8
Allele		
A	95	52.8
G	85	47.2
Haplotypes		
KRAS G13V & BRAF V600E		
GT	22	12.2
GA	26	14.4
AT	4	2.2
AA	128	71.1
KRAS G13V & EGFR exon20		
GA	45	25.0
GG	3	1.7
AA	50	27.8
AG	82	45.6
BRAF V600E & EGFR exon20		
TA	24	13.3
TG	2	1.1
AA	71	39.4
AG	83	46.1
BRAF V600E & KRAS G13V & EGFR exon20		
TGA	22	12.2
TGG	0	0.0
TAA	2	1.1
TAG	2	1.1
AGA	23	12.8
AGG	3	1.7
AAA	48	26.7
AAG	80	44.4

### PET-CT images: characteristics of the study population

**Fig. 1:**
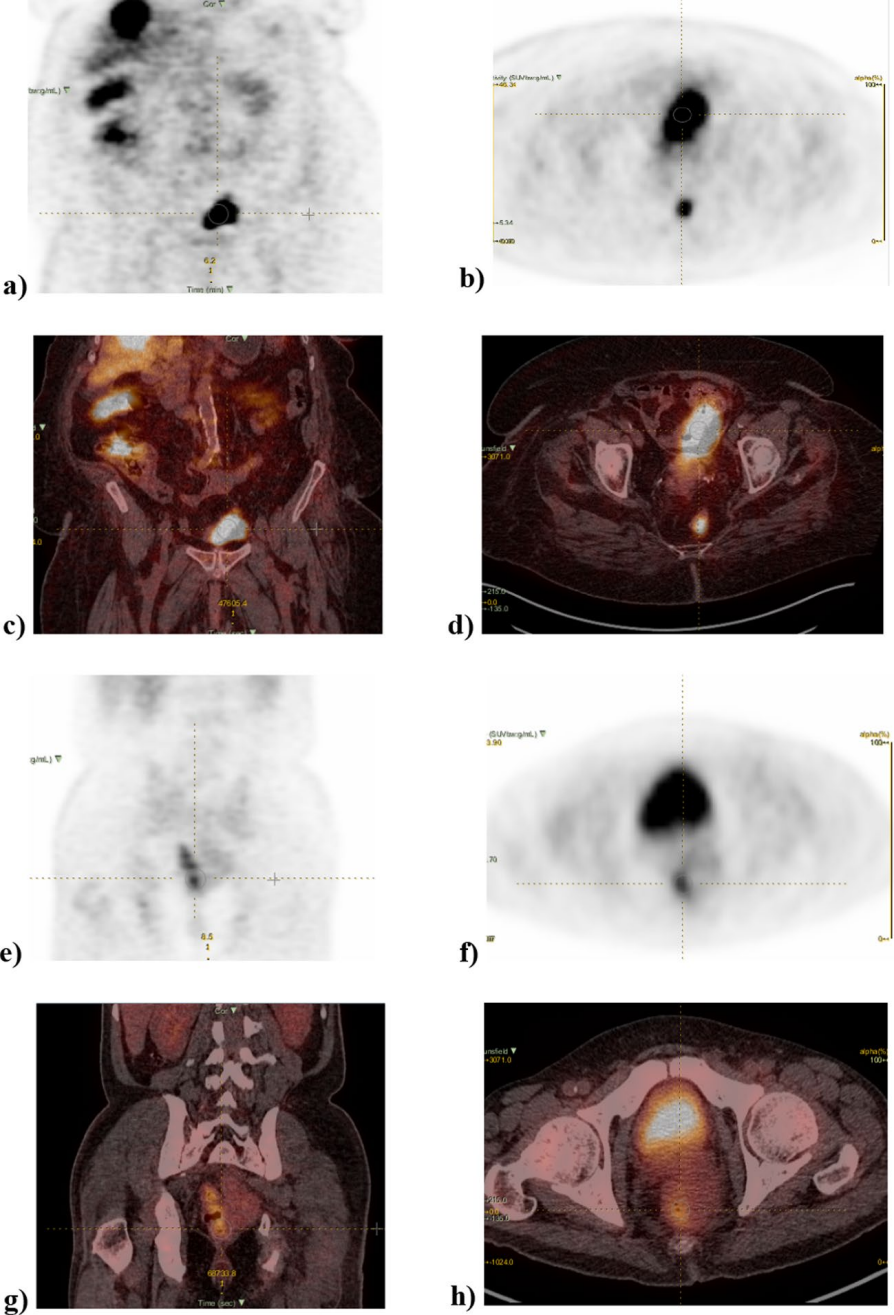
[^18^F]FDG PET image of an mCRC patient: **a** coronal PET image, **b** axial PET image, **c** coronal PET-CT fused image, and **d** axial PET-CT fused image of a 59-year-old female with rectal cancer with mutation (KRAS/EGFR) (SUV: 26.2; TLG: 278.5; TBR 2.6). **e** Coronal PET-CT image, **f** axial PET-CT image, **g** coronal PET-CT fused image, **h** axial PET-CT fused image of a 55-year-old male with rectal cancer with no mutation (SUV: 5; TLG: 114.3; TBR: 1.7)

The PET-CT image characteristics of mCRC patients, including the SUVmax, total MTV, total TLG, and maximum TBR, are illustrated in Table 2. The sites of metastasis according to the PET-CT images included the liver, lymph nodes, lungs, peritoneum, thyroid, bone, anal canal, and abdominal wall as shown in (Fig. 1).

**Table 2:** PET-CT images: characteristics of the study population

		Mean ± SD	Median (Min. – Max)
Primary and Metastatic Tumors	SUV max	13.44 ± 9.72	(4.0–46.30)
	SUV mean	7.44 ± 5.33	(2.0–30.20)
	Total MTV	395.0 ± 515.4	(22.37–2301.1)
	Total TLG	1626.7 ± 1926.5	889.7 (71.47–8451.4)
	Max. TBR	5.22 ± 3.61	4.03 (1.50–17.94)

*SD* Standard deviation

### Correlations between BRAF, KRAS, and EGFR genotypes and radiomic parameters

mCRC patients with the BRAF V600E mutation had a significantly greater SUV max, total TLG, and maximum TBR than did the patients in the heterozygous group (p < 0.001, = 0.002, and 0.014, respectively). A similar pattern was observed in patients with the KRAS G13V mutation, as these patients also had significantly greater SUV max, total TLG, and maximum TBR than did the patients in the heterozygous and wild-type groups (p < 0.001, = 0.004, and 0.001, respectively). For the exon 20 EGFR mutation, only the SUV max was significantly greater in mCRC patients with homozygous mutations than in those with heterozygous and wild-type mutations (p = 0.042). The total MTV did not significantly differ among patients carrying any of the three tested mutations as shown in Table 3.

**Table 3:** Correlations between BRAF, KRAS, and EGFR genotypes and radiomic parameters

	BRAF V600E (rs1133488022)			U	p
		TA (n = 26)	AA (n = 64)		
SUV max		7.0 ± 3.3	16.0 ± 10.3	262.00	< 0.001^*^
Total MTV		387.8 ± 473.7	397.9 ± 535.0	752.00	0.476
Total TLG		1150.8 ± 1572.1	1820.1 ± 2032.5	486.00	0.002^*^
Max. TBR		3.6 ± 1.8	5.9 ± 4.0	555.50	0.014^*^
	KRAS G13V (rs112445441)			H	p
	GG (n = 3)	GA (n = 42)	AA (n = 45)		
SUV max	5.1 ± 1.0	9.5 ± 6.3	17.6 ± 10.8	25.027	< 0.001^*^
Total MTV	98.3 ± 70.9	480.8 ± 610.7	334.7 ± 415.7	4.499	0.105
Total TLG	262.8 ± 198.7	1594.1 ± 2196.5	1748.0 ± 1693.2	11.151	0.004^*^
Max. TBR	3.3 ± 2.4	3.8 ± 2.1	6.7 ± 4.2	13.453	0.001^*^
	EGFR exon 20 (rs1050171)			H	p
	AA (n = 21)	AG (n = 53)	GG (n = 16)		
SUV max	9.6 ± 6.4	14.2 ± 10.5	16 ± 9.7	6.353	0.042^*^
Total MTV	269.5 ± 228.9	468.2 ± 624	317 ± 330.2	0.428	0.807
Total TLG	1200.2 ± 1746.7	1872.2 ± 2179.1	1373.4 ± 978	3.957	0.138
Max. TBR	4.5 ± 3.4	5.5 ± 3.8	5.1 ± 3.3	1.582	0.453

U: Mann–Whitney test

H: H for Kruskal–Wallis test

p: p value for Relationships between mutations and PET parameters

^*^: Statistically significant at *p* ≤ 0.05

### Correlations between the BRAF, KRAS and EGFR haplotypes and radiomic parameters

Assessment of the association of radiomic parameters with different haplotypes rather than with single mutations revealed that the SUV max was significantly greater in patients carrying the mutant alleles of all combinations of the studied mutations than in those with other possible haplotypes, including KRAS G13V-BRAF V600E (p < 0.001), KRAS G13V-EGFR exon20 (p = 0.004), BRAF V600E-EGFR exon20 (p = 0.038), and BRAF V600E-KRAS G13V-EGFR exon20 (p = 0.007). TLG and TBR were significantly greater in patients with the KRAS G13V-BRAF V600E haplotype alone (p = 0.008 and 0.006, respectively), and TLG was significantly greater in patients with the KRAS G13V-EGFR exon 20 (p = 0.023). However, both TLG and TBR failed to significantly differ among the other haplotypes as shown in Table 4.

**Table 4:** Correlations between radiomic parameters and genetic haplotypes

	No	SUV max		Total TLG		Max. TBR	
		Mean ± SD	p	Mean ± SD	p	Mean ± SD	p
KRAS G13V & BRAF V600E							
AA	128	15.2 ± 10.4	< 0.001^*^	1690.4 ± 1864.9	0.008^*^	5.74 ± 3.94	0.006*
Other	52	9.2 ± 5.9		1469.8 ± 2063.6		3.93 ± 2.14	
KRAS G13V & EGFR exon20							
AG	82	15.2 ± 10.1	0.004^*^	1734.3 ± 1841.8	0.023^*^	5.46 ± 3.60	0.237
Other	98	11.9 ± 9.1		1536.7 ± 1990.0		5.01 ± 3.61	
BRAF V600E & EGFR exon20							
AG	83	14.9 ± 10.3	0.038^*^	1641.7 ± 1828.4	0.189	5.37 ± 3.62	0.511
Other	97	12.2 ± 9.1		1613.9 ± 2006.5		5.09 ± 3.60	
BRAF V600E & KRAS G13V & EGFR exon20							
AAG	80	15.3 ± 10.3	0.007^*^	1691.1 ± 1844.2	0.058	5.45 ± 3.64	0.322
Other	100	12 ± 9		1575.2 ± 1988.3		5.03 ± 3.58	

*SD* Standard deviation

p: p value for Relation between SUV max, Total TLG, Max. TBR and Haplotype

^*^: Statistically significant at *p* ≤ 0.05

### Receiver operating characteristic (ROC) analysis of PET parameters

**Fig. 2:**
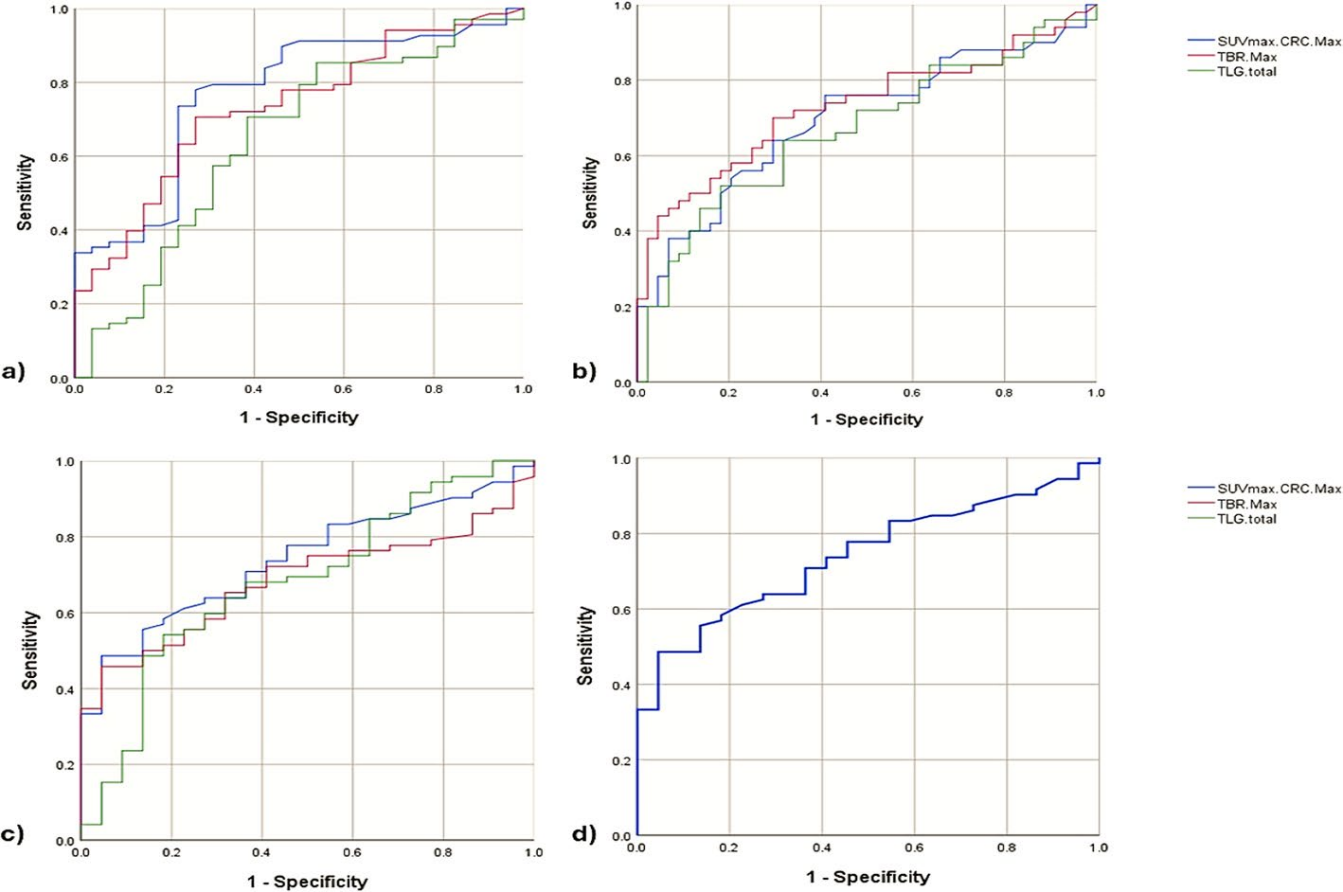
ROC Curve Analysis for Evaluating the Sensitivity and Specificity of PET Radiomic Parameters in Predicting **a** BRAF V600E, **b** KRAS G13V, **c** EGFR exon 20, and **d** the KRAS/BRAF/EGFR haplotype in mCRC patients

ROC analysis was used to investigate the sensitivity and specificity of the SUV max, TBR, MTV and TLG for predicting different mutations and the KRAS/BRAF/EGFR haplotype in mCRC patients as shown in (Fig. 2). Statistical analysis revealed that the SUVmax had 59.09% specificity and 76% sensitivity for the KRAD G13V mutation, 73.08% specificity and 77.94% sensitivity for predicting the BRAF V600E mutation, and 63.64% specificity and 70.83% sensitivity for the EGFR exon 20 mutation. For TBR, the results indicated 70.45% specificity and 70% sensitivity for the KRAS G13V mutation, 73.08% specificity and 70.59% sensitivity for the BRAF V600E mutation, and 55.56% specificity and 77.27% sensitivity for the EGFR exon 20 mutation. TLG showed 81.82% specificity and 52.0% sensitivity for the KRAS G13V mutation, 61.54% specificity and 70.59% sensitivity for the BRAF V600E mutation, and 77.27% specificity and 55.56% sensitivity for the EGFR exon mutation. For predicting the mutant haplotype KRAS/BRAF/EGFR, the SUVmax showed 63.64% specificity and 63.89% sensitivity with a cutoff value of 8.9 and an AUC of 74%.

## Discussion

One of the most important therapeutic approaches for MCRC is anti-EGFR targeting. It has been difficult for oncologists to integrate EGFR antibodies into the mCRC continuum of therapy for nearly 20 years. The mutational status of key genes is a highly significant predictor of the clinical outcomes of patients receiving anti-EGFR drugs (Doleschal et al. 2022). Although KRAS mutations in codons 12 or 13 have received much attention and have been linked with recommendations for patients receiving anti-EGFR treatment, other mutations, including BRAF V600E and EGFR exon 20, have also been linked to worse prognosis and poor clinical outcomes (Cutsem et al. 2016; Tsang et al. 2014). Nevertheless, due to the difficulty of obtaining samples for genetic testing without surgery or biopsy, the high cost of liquid biopsy testing, and the difficulty in detecting distant metastases, noninvasive markers that can predict the mutational status of mCRC patients are needed. Radiomics is a promising approach that utilizes some features of tumor imaging to reflect some genetic characteristics of the disease and provides a potential noninvasive procedure to predict underlying molecular changes and, in some cases, to predict treatment outcomes and patient prognosis (Limkin et al. 2017). This study also showed that PET metabolic parameters contribute to the noninvasive prediction of BRAF, KRAS, and EGFR mutation status in mCRC.

The results of the current study indicated that approximately 70% of the enrolled mCRC patients carried KRAS exon 13 mutations, which is consistent with the findings of previous studies(Mesti et al. 2023; Peeters et al. 2013). KRAS mutation is a well-established biomarker for resistance to anti-EGFR monoclonal antibody treatment in metastatic CRC. The prognostic usefulness of KRAS mutations in CRC, on the other hand, remains controversial. KRAS mutations have been associated with a poorer prognosis in certain studies, but not in others (Won et al. 2017). The BRAF V600E mutation was also prevalent in approximately 50% of the studied mCRC patients. The BRAF V600E mutation is regarded as a poor prognostic and predictive biomarker and is typically associated with short disease-free survival and overall survival times, as well as poor treatment response (Guerrero et al. 2022). Recently, therapeutic approaches developed to inhibit BRAF in multiple myeloma patients have shown success, and similar approaches are being investigated for colorectal cancer patients (Ros et al. 2021).

Statistical analysis revealed significantly greater SUV max, TBR, and TLG in patients carrying both mutations than in patients in the wild-type group; however, MTV was not significantly associated with any of the studied mutations. These results are consistent with previous reports associating increased predictability of KRAS and BRAF mutations with metabolic PET parameters in mCRC; for example, both the SUVmax and TLG were significantly greater in the KRAS/BRAF-mutated group than in the wild-type group (He, et al. 2021).

EGFR exon 20 mutation had the lowest incidence, and its carriers only had a significantly elevated SUV max, while the other metabolic parameters were not significantly elevated. The SUVmax of the primary tumor could indicate the EGFR status. EGFR mutations are involved in tumorigenesis, tumor progression in CRC and the development of resistance to various treatments. By blocking mAb binding, somatic EGFR sequence alterations, such as those in the extracellular domains of the EGFR-mAb interaction interface (G465R, G465E, S468R, and S492R), confer resistance to therapeutic agents, including cetuximab and panitumumab (Bertotti et al. 2015; Price et al. 2020). Furthermore, in the presence of cetuximab, R198/R200 methylation and a mutation in the EGFR kinase domain (V843I) are linked to disease progression (Liao et al. 2015).

Haplotype analysis revealed an association between elevated metabolic PET parameters, especially the SUV max, and KRAS/BRAF/EGFR mutations in mCRC patients. The SUV max was significantly different between patients with KRAS/EGFR/BRAF mutations and those with other genotypes, suggesting that the SUV max may be useful for predicting the KRAS/EGFR/BRAF mutational status in mCRC patients with high-to-moderate sensitivity and specificity at a cutoff value of 8.3. These results are in accordance with previous studies that have shown ROC curve analysis for the SUVmax of the primary tumor for predicting the efficacy of KRAS/NRAS/BRAF mutations in CRC (He et al. 2021). One significant limitation of our study is the potential impact of treatment status on [^18^F]FDG uptake, as various therapies such as chemotherapy and radiation can alter metabolic activity, thus affecting [^18^F]FDG imaging results (Johnstone et al. 2020; Boellaard et al. 2015). This treatment-induced variability may obscure the distinction between disease progression and therapeutic response (Evangelista, et al. 2021; Okuyama et al. 2023). To enhance the accuracy of [^18^F]FDG imaging, future research should focus on longitudinal studies to monitor changes in [^18^F]FDG uptake in relation to different treatments(Kung, et al. 2019), comparative studies to understand the effects of various therapies and investigations into the underlying mechanisms driving these changes, Additionally, exploring alternative imaging modalities less affected by treatment status could provide complementary diagnostic insights (Casali, et al. 2021), while developing standardized imaging protocols could improve the reliability and comparability of [^18^F]FDG PET studies across diverse clinical settings(McDougald et al. 2020).

### Study limitations

The present study is subject to several limitations that that merit consideration. The sample size was small, involving only 90 patients from a single institution, which limits generalizability. The analysis focused on only three genetic mutations (KRAS, BRAF, EGFR), despite colorectal cancer’s complexity with many potential driver mutations. The cross-sectional design assessed mutation status and PET-CT parameters at one point in time, lacking longitudinal data on disease progression. The study was conducted solely in an Egyptian population, raising questions about applicability to other groups. Multivariate analysis was also absent, which could have provided more comprehensive insights. Despite these limitations, the study offers preliminary evidence for using 18F-FDG PET-CT radiomic parameters, particularly SUVmax, as non-invasive biomarkers for mutation status prediction. Future research should address these limitations to strengthen the findings.

## Conclusion

The present study underscores the significance of [^18^F] FDG PET-CT radiomic parameters, particularly SUVmax, in their association with KRAS, BRAF, and EGFR mutations in metastatic colorectal cancer patients. These findings suggest that SUVmax may serve as a valuable noninvasive predictive biomarker. Haplotype analysis further demonstrates that SUVmax can effectively differentiate patients with these mutations, exhibiting both high specificity and sensitivity.

## Recommendations

We recommend that future research systematically incorporate clinical variables, such as TNM staging and treatment status, as these factors significantly influence FDG uptake and radiomic parameters. Employing both univariate and multivariate analyses, in addition to ROC analysis, is essential to enhancing the robustness of study findings. The establishment of standardized imaging protocols is also vital for ensuring consistency across different studies. Longitudinal investigations should be pursued to assess the impact of evolving clinical variables over time. Furthermore, exploring additional relevant factors and fostering collaboration with clinical teams will be key to improving predictive models for KRAS, BRAF, and EGFR mutations. Finally, emphasizing the inclusion of diverse patient cohorts will enhance the generalizability of the results and strengthen predictive capabilities in oncology research.

## Data Availability

An email to the corresponding author is available upon request.

## Abbreviations

BRAF: V-Raf Murine Sarcoma Viral Oncogene Homolog B1
CRC: Colorectal cancer
EGFR: Epidermal Growth Factor Receptor
ERK: Extracellular signal-regulated kinase
FDG: Fluorodeoxyglucose
KRAS: Kirsten Rat Sarcoma Viral Oncogene Homolog
MAPK: Mitogen-activated protein kinases
mCRC: Metastatic colorectal cancer patients
MEK: Mitogen kinase
mTOR: Mammalian target of rapamycin
MTV: Metabolic Tumor Volume
PET-CT: Positron Emission Tomography-Computed Tomography
PI3K: Phosphatidylinositol-3-kinase
RAS: Rat Sarcoma Virus
ROC: Receiver operating characteristic
SNPS: Single nucleotide polymorphisms
SUV: Standard Uptake Values
TBR: Tumor-To-Background Ratios
TLG: Total Lesion Glycolysis
